# A qualitative evaluation of a stress management programme for HIV and AIDS home-based care workers in Tshwane, South Africa

**DOI:** 10.1080/17290376.2020.1810747

**Published:** 2020-09-13

**Authors:** P. M. Kupa, L. S. Geyer

**Affiliations:** Department of Social Work & Criminology, University of Pretoria, Pretoria, South Africa

**Keywords:** Acquired Immune Deficiency Syndrome (AIDS), Human Immunodeficiency Virus (HIV), HIV and AIDS home-based care workers, South Africa, Stress management programme, Tshwane metropolitan municipality

## Abstract

The HIV and AIDS pandemic resulted in increased demands on the South African healthcare system and contributed to elevated stress levels among healthcare workers, including home-based care workers. The goal of the study was to evaluate a stress management programme for HIV and AIDS home-based care workers in Tshwane, South Africa. Social constructionism was adopted as the theoretical framework of the study. The study implemented intervention research and adopted a qualitative research approach, specifically the instrumental case study. Non-probability sampling, specifically volunteer sampling was utilised to recruit a group of twelve HIV and AIDS home-based care workers (*n* = 12). The data were collected through semi-structured interviews and administered before and after exposure to the stress management programme. The research findings, based on thematic analysis, revealed that the programme was effective in mitigating the impact of stress experienced by the HIV and AIDS home-based care workers in Tshwane. Recommendations are proffered for the refinement of the newly developed stress management programme for implementation among HIV and AIDS home-based care workers in similar field settings.

## Introduction

The Human Immunodeficiency Virus (HIV) and Acquired Immune Deficiency Syndrome (AIDS) pandemic have been around for more than three decades now. Progress has been made in containing the pandemic, but there are still challenges to be overcome (Heunis, Wouters, & Kigozi, 2012; Okeke, 2016). The Joint United Nations Programme on HIV and AIDS (UNAIDS) reported that eastern and southern Africa remain heavily affected by HIV, accounting for an estimated 19.4 million of the 36.7 million of people living with HIV worldwide as at the end of 2017 (Joint United Nations Programme on HIV and AIDS Fact sheet, July 2018). It is further reported that the epidemic continues to be a concern in Southern Africa, with South Africa being home to an estimated 7.1 million people living with HIV (Joint United Nations Programme on HIV and AIDS – South Africa, 2018).

As households in South Africa became affected by HIV and AIDS, there was a greater need for care and support. With the shortage of professional healthcare practitioners, the greater burden of care for people living with HIV and AIDS remained with informal community-based carers (Majaja et al., 2009). Marais (2005) posits that the post-1994 overhaul of South Africa’s healthcare system was a bid to ensure that ‘care in the community’ became ‘care by the community’. As such, community-based care became part of the ‘continuum of care’ which linked together the various levels and zones of the public health care system and other role players to provide an integrated service that addressed the basic needs of people infected with or affected by HIV.

Over the last decade, the National Department of Health allocated funds for the comprehensive response to HIV and AIDS. Non-governmental and community-based organisations in the HIV and AIDS field which trained and employed community and home-based workers were among the main beneficiaries of this allocation. Consequently, a large grouping of lay health workers emerged. In addition, the government introduced the umbrella term ‘community health worker’ for all lay community health workers and adopted a policy framework for their training and remuneration in line with the National Department of Health Community Health Workers Policy Framework (2004) (Schneider, Hlophe, & Van Rensburg, 2008).

In order to give background to the National Department of Health initiative of community health workers, it is important to highlight that it is linked to the Expanded Public Works Programme (EPWP). The EPWP is a project of the National Department of Public Works that aims to avail work opportunities for the unemployed from poor households. The programme employs workers on a temporary or on-going basis, either by government departments, contractors or non-governmental organisations. The EPWP creates work opportunities through four sectors, namely: infrastructure, non-state, environment and culture and social sectors. For many, the programme provides the much-needed work experience, acquisition of relevant work skills and validates their ability to contribute to their communities (National Planning Commission, 2011; Welcome to EPWP, 2013). Of relevance to the study reported here, is the social sector. The social sector provides work opportunities for people to deliver social development services, which include home and community-based care. The overall coordinator of the sector is the National Department of Social Development with the National Department of Health taking direct responsibility for the home and community-based care (National Planning Commission, 2011; Welcome to EPWP, 2013).

Home-based care workers (HBCWs), like other healthcare practitioners, found themselves working with the most challenging health problems, in circumstances where poverty and hopelessness are prevalent. The potential for demotivation is high given the fact that often there is little recognition for the work they do. It is sometimes referred to as the ‘dirty work’ which is not regarded as real work and thus undermined, whilst they render a much-needed service to those on the margins of society (Rohleder & Swartz, 2005). Efforts to have formal support structures for the HBCWs were recommended but were quite patchy. In the meanwhile, they had to continue working in strain and hardship (Campbell & Foulis, 2004; Campbell, Nair, Maimane, & Sibiya, 2008).

Studies have revealed that carers experienced the work as emotionally demanding and draining and thus affecting their mental health. Some HBCWs reported that they found the work exhausting, overwhelming and stressful to the point of having thoughts of leaving the job. Some commented about the guilt and despair they experienced when they had to work within the poor living conditions that some of their patients live in. The ever-increasing workload in spite of the availability of, amongst others, antiretroviral drugs was also a concern as the HBCWs worked in communities where poverty was prevalent. HBCWs knew their main role as depending on the client’s needs, to engage in lay counselling, teach family members on how to care for the patient, do simple nursing tasks like dressing of wounds, monitor medication compliance, provision of food parcels, do ordinary household chores and render palliative care to those who needed it. In addition, they had to link the patients with comprehensive social and health support services that were available to the community (Lund & Budlender, 2009; Orner, 2006; Wringe, Cataldo, Stevenson, & Fakoya, 2010). In practice, they found themselves being compelled to do more (Dageid, Sedumedi, & Durkert, 2007; Hlophe, 2006; Orner, 2006).

Furthermore, situations of high poverty levels have been found to compromise the effective management of HIV infection and disease progression (Linganiso & Gwegweni, 2016; Pienaar, Van Rooyen, & Walsh, 2017). HIV and AIDS is multifaceted and subsequently require numerous duties from HBCWs employed within the specific context. The duties include, amongst others, the following: mediator between clinic and patients; caring for, often bedridden, patients at their home; supporting the significant others and extended family of patients; the treatment of symptoms associated with AIDS; in the event of a patient’s death, assistance with the burial process and bereavement counselling (Lekganyane & Alpaslan, 2019). Hence, the study reported on in this article focused specifically on HBCWs who worked within the context of HIV and AIDS care to ensure the designed programme is tailored for their unique working conditions and addressing their specific needs for stress management.

Symptoms associated with HIV and AIDS-related stress and burnout may be physical (for example, headaches), behavioural (for example, irritability) or cognitive and emotional (for example, pessimism and sadness) and physiological (for example, unstable blood pressure, ulcers, muscle tension and headaches) (Cogan, Klein, Magongo, & Kganakga, 2005; Colligan & Higgins, 2006; Tanyi, Pelser, & Okeibunor, 2018). The employees often remain in such stressful working conditions as they perceive the work as a potential avenue for better jobs (Lund, 2010).

Findings from a study in Tshwane among HIV and AIDS lay counsellors (which is also applicable to HBCWs), emphasised the need for a structured workplace support programme to assist them to cope with the psychosocial challenges of the work as there were none available (Kabamba, 2009). A similar study in the Free State Province (one of the nine provinces in South Africa, situated in the centre of the country) reported that lay healthcare workers (HBCWs included) complained to nurses about the fact that they perceived their psychological wellbeing as neglected and therefore requested some form of workplace support (De Wet & Du Plooy, 2012). From the findings of these studies it was recommended that in order to meet the psychosocial needs of HBCWs working with people living with HIV and AIDS, stress relief therapies have to be improved and care of the caregiver programmes emphasised, because carers continued to be overwhelmed by the practical and emotional demands of care work in spite of available support services (Geteri & Angogo, 2013; Lekganyane & Alpaslan, 2019; Majaja et al., 2009; Orner, 2006).

From the aforementioned, it became evident that there is a need for a workplace support programme targeted at HBCWs to manage, among others, stress. In order to address the stress-related workplace support needs of the HBCWs, the authors worked with HBCWs from previously disadvantaged communities in the Tshwane Metropolitan area because of the following reasons:

- Literature and research consulted indicated that in spite of the need for a workplace stress management programme for HIV and AIDS HBCWs, there were a few existing programmes specifically for this target group.
- Secondly, although there has been a similar research project carried out nationally by the Human Sciences Research Council, Tshwane was not included in the sampling process.
- Finally, the researcher targeted non-governmental organisations in Tshwane working with people living with HIV and AIDS. The HBCWs working for these organisations and their supervisors/managers were recruited as research participants. Their inputs facilitated the identification of stress-related support needs in their area of work. The sourced inputs led to the design of the stress management programme.

The main goal of the study reported on here was, therefore, to evaluate the newly developed stress management programme for HIV and AIDS HBCWs in Tshwane.

In addition to the introduction, this articles offers a brief overview of social constructionism, as a theoretical framework underpinning the programme design and evaluation, an overview of the stress management programme, followed by methods, discussion of research findings, comparison of pre- and post-exposure data as well as conclusions and recommendations.

## Social constructionism

Social constructionism is explained as one of the broad schools of thought in the social sciences and draws its influence from a number of disciplines, including philosophy, sociology and linguistics, making it multi-disciplinary in nature (Burr, 2003; Lock & Strong, 2010). As a result, it has been associated with the post-modern era qualitative research (Andrews, 2012). The approach, according to Freedman and Combs (as cited in Botha, 2002), encourages looking at reality from a different perspective to challenge common sense knowledge about the world we live in.

Social constructionism proposes that there are no ‘universal truths’; reality or truth is defined by participants/service users and this is time and context specific (Lock & Strong, 2010). Furthermore, Rosenthal and Peccei (as cited in Cunliffe, 2008, p. 127) add that its notion of the importance of subjective reality allows researchers to discover how the participants make sense of their social settings and negotiate collective meaning, as well as the impact of that collective meaning within the broader social context. Social constructionism further proposes that realities are maintained and organised through stories (Freedman & Combs, 1996). In striving to make sense of life, people arrange their experiences of events in sequences across time in such a way as to arrive at a coherent account of themselves and the world around them. The success of this ‘storying’ of experience provides them with a sense of continuity and meaning in their lives (Freedman & Combs, 1996).

Through social constructionism, the HBCWs were given the opportunity to ‘tell their story’ without the authors giving their own views of what they might be articulating. The approach thus provided a platform from which the HBCWs were able to express their subjective perceptions of their world of work and the challenges they faced. As the research process unfolded, the HBCWs had the opportunity to ‘author’ the stress management programme and also provide valuable inputs during the evaluation thereof.

## Overview of the stress management programme

In an effort to gain insight into the workplace support needs of the HBCWs, the authors consulted existing literature and research studies on HIV and AIDS home-based care and also on successful stress management programmes in the healthcare sector (Hatzipapas, 2013). In addition to studying literature, empirical evidence was sought from the HBCWs themselves (in Tshwane) through a needs assessment process. The findings of the process confirmed that there was a need for a stress management programme for HBCWs, addressing the following areas of priority: coping with emotional stress, coping with patient care and availability of regular and structured debriefing sessions (Kupa, 2019).

The stress management programme is an interactive programme with the goal to equip the HBCWs with skills and knowledge to cope with workplace stress, strengthen existing coping mechanisms and promote resilience in the face of stressful working conditions. The evaluation of the programme was group-based and facilitated with twelve HBCWs (research participants). Such a group size allowed healthy development of group cohesion, promotes active participation, sharing of ideas and opinions about the programme with limited room for cliques resulting in overall participant satisfaction (Greeff, 2011; Wheelan, 2009).

The programme consisted of seven sessions covering five modules with each session’s duration of about 90 min. The programme ran over seven Monday mornings and took seven weeks to complete from February to March 2018. Each session started with an ice-breaker to assist the participants to de-role and focus on the topic of the day and ended with a written session evaluation. The programme contents are briefly outlined in Table 1.

**Table 1.** Brief contents of the stress management programme.

Session/Module	Objectives	Content	Activities
Module 1: Introduction to the programme Session 1: Background information on the programme	Explanation of the background and purpose of the programme. Familiarising participants with the programme schedule.	Interactive discussion on: description of home-based care (HBC); current HIV and AIDS statistics, workplace stress in HIV and AIDS HBC; and appraisal of the content and sequence of sessions by participants.	Session started with an ice-breaker followed by an interactive discussion of the session content. Class activity 1 was used to brainstorm the realities around HBC. Class activity 2 focused on the current statistics on HIV and AIDS in relationship to the daily work of HBCWs. Class activity 3 explored workplace stress.
Module 2: Understanding illness Session 2: The experience and meaning of illness to the patient	Familiarise participants with the concepts of health, illness and disease. Gaining insight into the experience of illness to the patient and family. Understanding the role of HBC in working with the patient and family.	Interactive discussion on: the explanation of terminology; the impact of illness on the family and reactions; the impact of illness on the family; and the role of HBC worker in working with chronically ill patients using BREAKS^a^ protocol.	Session started with an ice-breaker followed by an interactive discussion of session content. Class activity 1 explored patient reactions to illness, Class activity 2 ensured the practical application of the BREAKS protocol.
Module 3: Understanding stress Session 3: What is stress?	To understand stress, broadly. Gaining insight into stressors in HIV and AIDS HBC.	Interactive discussion on: description of stress; individual responses to stress; and exploration of what could be done to manage workplace stress.	Ice-breaker used to start the session followed by interactive discussion of content of the session. Class activity 1 focused on what stress is – the phenomenon. Class activity 2 focused on responses to stress.
Module 4: Stress management techniques Session 4: Rational emotive-behavioural therapy	To understand cognitive behavioural techniques as a stress management strategy. To be able to apply the techniques in work life.	Interactive discussion on: what is cognitive behavioural therapy (CBT); an introduction to the ABCDE^b^ model of CBT.	Session started with an ice-breaker followed by interactive discussion of session content. Class activity 1 explored the understanding of CBT. Class activity 2 encompassed the practical application of the ABCDE model.
Session 5: Critical Incident Stress Debriefing (CISD)	To understand CISD as a stress management strategy.	Interactive discussion on: trauma and its relationship to stress; an explanation of the seven phase model of CISD.	Session started with an ice-breaker followed by interactive discussion of session content. Class activity 1 focused on the understanding of trauma. Class activity 2 focused on the practical application of CISD.
Session 6: Mindfulness-based stress reduction (MBSR)	To understand MBSR as a stress management strategy. To be able to apply the strategy in work life.	Interactive discussion on: description of MBSR with a special focus on body scan and meditation techniques.	Session started with an ice-breaker followed by interactive discussion of session content. Class activity 1 focused on what MBSR entails. Class activity 2 was the practical application of two techniques of MBSR.
Module 5: Building resilience Session 7: Road to resilience	Understanding of resilience and how it can be promoted. Identification of available support networks to manage stress.	Interactive discussion on: what is resilience; what can be done to promote it; and ways to build resilience.	Session also started with an ice-breaker followed by interactive discussion of session content. Class activity 1 focused on an understanding of resilience. Class activity 2 focused on support systems in the management of stress. The session concluded with a candle lighting exercise.

^a^BREAKS protocol is used to ‘break bad news’ to patients, such as an HIV–positive diagnosis or terminal illness. The acronym stands for B – Background, R – Rapport, E – Exploring, A – Announce, K – Kindling, and S – Summarising (Narayanan, Bista, & Koshy, 2010).

^b^A technique within CBT to challenge patients’ irrational thoughts and the cause(s) thereof. The acronym stands for A – Activating event, B – Belief one has about the event, C – Consequences, D – Disputing the irrational thought, and E – a new Effective way of thinking/or approach a situation (Choudhury, 2013).

The programme was facilitated through group work methods that are specific to working with adults and allowed HBCWs to openly share their views of reality and challenge with one another and in the process gain new insights, develop a group identity and achieve mutual understanding over time. Furthermore, the process was participant-centred and both the facilitator (the first/lead author) and participants related to one another as adults (Greeff, 2011; Gregory & Thorley, 2013; Jordan, 2011).

## Method

Intervention research (IR), specifically the sub-type Design and Development, was operationalised to design, implement and evaluate the stress management programme (Thomas & Rothman, 1994). Although Design and Development consist of an integrated model of six phases, this article focuses only on Phase 4, that is, early development and pilot testing (Thomas & Rothman, 1994). An overview of the entire IR process is beyond the scope of this article. This article focuses exclusively on Phase 4 of the IR process within a greater study. As such, this article reports on the stress management programmes and its qualitative evaluation results.

A qualitative research approach was adopted for the study, specifically the instrumental case study – the HBCWs in Tshwane (Fouché & Schurink, 2011). The value of this qualitative design was that it enabled gathering descriptive and comprehensive data from the participants to capture their views, opinions and perspectives on how they experienced the stress management programme and to solicit their recommendations for its refinement (Campbell, 2014).

### Participants

The population consisted of all HIV and AIDS HBCWs in Tshwane working for non-governmental and community-based organisations. The National Norms and Minimum Standards for Home and Community Based Care and Support Programme (Department of Social Development [DSD], 2007) states that all non-governmental and community-based organisations rendering HIV and AIDS home-based care must have a minimum of ten home-based care workers and one manager. Furthermore, the Gauteng HIV-related Services Directory (2012) cites approximately 30 non-governmental and community-based organisations rendering HIV and AIDS home-based care distributed over six regions of the Tshwane Metropolitan. The population was therefore estimated at approximately 300 HIV and AIDS HBCWs.

Non-probability sampling techniques were used to recruit participants for the study. Purposive sampling was used to select HBCWs in the employ of an organisation in one region of Tshwane to participate in the study. The limitation of this type of sampling is that it is prone to researcher subjectivity and bias (Etikan, Musa, & Alkassim, 2016). The purposive selection (sampling) of the organisation was based on the following criteria: it had to have been in operation for at least two years in the HIV and AIDS home-based care field; registered with the DSD as a not-for-profit organisation; have a functioning Board of Management; and, a minimum of eight home-based care workers. Volunteer sampling was used to recruit HIV and AIDS home-based care workers in the employ of the organisation to participate in the pilot testing/evaluation of the stress management programme (Padgett, 2017). The disadvantage of this sampling technique is that it is usually subject to volunteer bias (Sharma, 2017). Prospective participants had to have worked as HIV and AIDS home-based care workers for at least one year, irrespective of age and gender.

Twelve participants were successfully recruited and participated in the study. All the participants identified themselves as African Black and female. The ages of the participants ranged from 21 to 43 years of age at the time of the study, with the average age being 30.6 years. With regards to marital status, only two participants indicated that they were married, whilst the rest reported being single. Eight participants had attained a Grade 12 certificate, one had a post-Grade 12 qualification, whilst only three did not have a Grade 12 certificate. With regards to years of experience as HBCWs, there were four participants whose years of experience ranged from 1 to 2, whilst the remaining eight participants’ ranged from 5 to 7 years of experience. The demographic profile of the participants is compatible with that of other HBCW’s in similar studies conducted in South Africa and sub-Saharan Africa (cf. Lehmann & Sanders, 2007; Lekganyane & Alpaslan, 2019; Lund, 2010; Ntobeng, 2016).

### Data collection and analysis

The qualitative data were collected through two semi-structured interviews with interview guides. The first schedule, administered before exposure to the programme, had twelve open-ended questions. The aim of the interview schedule was to gather information mainly on the workplace challenges the participants faced, including stress, coping mechanism used, if they were coping in the first place, and what their expectations were regarding the stress management programme. The second interview schedule with nine questions was administered after exposure to the programme. It focused on the participants’ workplace challenges and coping mechanisms, the only difference was that it asked participants if their pre-exposure expectations were met and requested suggestions for improvements to the programme (see Appendix). The before and after programme exposure interviews were conducted by the first/lead author, including the programme delivery. Qualitative data gathered from the semi-structured interviews were analysed through a process of thematic analysis as proposed by Braun and Clarke (2013). The authors ensured the trustworthiness of the qualitative study through credibility, dependability, and confirmability. The authors managed the risk of research reactivity and bias by utilising observer triangulation, member checking and peer debriefing (Lietz & Zayas, 2010; Shenton, 2004). The authors also consulted with colleagues and other professionals knowledgeable in the field of healthcare throughout the entire research process (i.e. peer debriefing), while confirming the findings with a selected number of participants (i.e. member checking). The first author and an independent coder analysed the data and a consensus meeting confirmed the themes and sub-themes to be reported (i.e. observer triangulation).

### Ethical considerations

Ethical clearance (Ref no.: GW20150515HS) was obtained from the Research Ethics Committee of the university. The participating NGO also granted permission for the study to be conducted. Ethical considerations, such as written informed consent, no harm, confidentiality, no deception, were observed (Babbie & Mouton, 2011).

## Findings and discussion

The data were gathered from pre- and post-exposure to the stress management programme. Four themes with sub-themes were extracted from the pre-exposure data and another four themes with sub-themes were extracted from the post-exposure data. Where HBCWs expressed themselves in a language other than English, the translated quotations are presented. A discussion of the themes starts with the pre-exposure data followed by the post-exposure data. This section ends with a comparison of the two sets of data.

### Themes and sub-themes from the pre-exposure data

The four themes and accompanying sub-themes that were extracted pre-exposure to the programme, follow.

### Theme 1: job fulfilment indicators

Within this theme, the following three sub-themes were identified among the responses of the participants: professional growth opportunities, enjoyable work and opportunity to care for others in need. The sub-themes demonstrated the differing views that the participants had about what fulfilled them in their work as HBCWs. In line with social constructionism, all their inputs had to be considered to ensure that their views of social reality are accurately captured as there was no view that is better than the other (Gergen, 2001).

### Sub-theme 1.1: professional growth opportunities

The majority of the participants explained that although challenging, the work provided them with opportunities to stretch themselves and deal with situations that they would not ordinarily deal with if they were not in this field of work. In addition to the work experience they gained, the organisation also made an effort to offer opportunities to attend various short courses and workshops organised for NGOs to acquire additional skills relevant to their work. The sentiment was captured by one of the participants in this manner:

I like my job because I learn new things every day. It gives me a lot of opportunities … sometimes they take us to courses … courses offered by Social Development related to psychosocial … I get challenges every day and I feel like I am achieving something.

Another participant was more specific in terms of the learning opportunities:

I like the way we work … we get knowledge … they teach us everything. We do not only get training from Social Development, but we are also taught about health-related matters from the clinic.

Apart from providing unemployed women with job opportunities, the EPWP social sector plan’s goal is also to ensure that participants receive training and work experience that could assist them to compete in the open labour market (Department of Social Development, Department of Education, & Department of Health, 2004). The participants’ responses bore testimony to the fact that the programme was upgrading their skills and they were appreciative of the opportunity.

### Sub-theme 1.2: enjoyable work

A few participants’ views were that they found their work enjoyable and that is what kept them motivated on a daily basis. Participants expressed their experience by stating that:

I enjoy working with people … I actually enjoy working with children the most.

I enjoy working with children … I did not know how to work with children before I was a home-based care worker. I also enjoy caring for ill people.

A South African study and a review of altruism and volunteerism confirmed that working with people living with HIV and AIDS had been frequently cited as a ‘labour of love’ by carers (Du Preez & Niehof, 2008; Haski-Levennthal, 2009). Carers engaged in this kind of work presumably for the intrinsic rewards and the good feeling about themselves that they derived from the work.

### Sub-theme 1.3: opportunity to care for those in need

One participant indicated that what she found satisfying about the work was that she got the chance to help those in need. She expressed it as follows:

I like to help children and their families, especially those who have lost hope, and those who have family members that are ill.

The participant’s view of her work could be seen as altruism at it appeared that she found fulfilment in helping those in need without expectation of any extrinsic reward (Haski-Levennthal, 2009).

The three sub-themes discussed so far indicated that the participants found their work stimulating, and contributing in some ways to personal or professional growth and fulfilment. No participant mentioned that they did not find their work satisfying.

### Theme 2: participants’ perceptions of challenges in the execution of their work

Three sub-themes were extracted from the interviews, namely: lack of material resources that affect the ability to meet patients’ needs, the experience of work as emotionally draining and lack of skills to deal with challenging patients. The theoretical framework encourages looking at reality from different perspectives to challenge common sense knowledge about the world we live in (Freedman & Combs, 1996 as cited in Botha, 2002). The authors, therefore, had to consider the views of the participants and not make assumptions from the needs assessment findings and literature consulted on the workplace challenges of home-based care.

### Sub-theme 2.1: lack of material resources that affect the ability to meet patients’ needs

The participants’ responses were varied with regards to this sub-theme. In spite of that, there were two main concerns pertaining to lack of material resources that they raised, namely lack of transport to home visits and lack of food parcels for impoverished families. The majority of the participants complained of having to walk long distances from one house to the other. They reported that in the communities that they work in, the houses were far apart. A participant expressed her concern in the following manner:

In this kind of work, we walk long distances because there is no transport offered by the organisation. When you arrive at home you are tired and you do not have time for your children … the children need your time and attention. Houses in [name of the area removed] are far apart. You have to walk quite a distance to get to the next patient’s home … this you do in the hot sun.

Another participant said the following about lack of food parcels:

… sometimes during visits, I will get to a family that has no food at all … you find that there is nothing I can help with. When I have a meal at home, I think of them and I am overcome with pity. I ask myself if they do have anything to eat.

A South African study and another in Tanzania confirmed the concerns of the participants that they were sometimes expected to walk from one household to the other in the communities that they worked in (Greenspan et al., 2013; Lund & Budlender, 2009). It has also been found that there were situations where the HBCWs relied on their families for material support such as food and transportation in the execution of their duties as the NGOs they were contracted to, could not provide such essential support (Greenspan et al., 2013; Lund & Budlender, 2009). These were some of the constraints within which the participants had to work.

### Sub-theme 2.2: experience of work as emotionally draining

All participants had to give specific input in terms of whether they found their work challenging on an emotional level. All participants indicated that they found their work emotionally draining and the following responses were received as explanations:

Sometimes I am emotionally affected by the problems of my patients. I once had a patient that is physically disabled. She was staying with her mute boyfriend. She could not explain what help she needed. I could not communicate with the boyfriend to at least find out how I could help. I did not know where to start or what to do and left feeling frustrated … 

When you have been unable to help a patient, by the time you get home you are irritable with your own family. Sometimes you even end up not wanting to do home visits the next day.

At least two South African studies confirmed that home-based care in the HIV and AIDS field was emotionally and psychologically stressful and also physically burdensome by its nature (Akintola, 2011; Ntobeng, 2016). It appeared then that what the participants were attesting to was somehow expected given the reality of the kind of work they are engaged in.

### Sub-theme 2.3: lack of skills to deal with challenging patients

HBCWs are lay healthcare workers and therefore the DSD (2007) had prescribed that, on joining the programme, employing organisations must provide induction of at least three days within a month and thereafter twenty-four days of on-going training per annum. Organisations are also expected to arrange accredited training for them in home and community-based care. At face value, it appears that all these control measures should ensure that HBCWs are adequately prepared for what they have to deal with in patients’ homes. The participants’ responses seem to paint a different picture. Some of the responses were:

Sometimes I come across a family that is similar to mine. We grew up as orphans, so when I come across families like that, I think about my own situation and I struggle to address whatever is bothering them as I end up being overly sympathetic, I feel overwhelmed and not sure what to do.

Sometimes you get a family with such serious problems and you begin to think that your own personal problems are nothing as compared to theirs. You wish you could help them but the needs are just too much … it is beyond my abilities as a home-based care worker … in such cases I refer to other resources or to our social worker. At least she [social worker] is able to be of more help to them than me.

Another two South African studies have alluded to the fact that HBCWs, similar to the participants, complained that their on-going training and education had been neglected leading to insufficient knowledge and skills. They needed to improve their work skills regularly to stay relevant and capable to service the variety of needs that their patients presented with and also be able to know when and where to refer (Hlophe, 2006; Lehmann & Sanders, 2007).

### Theme 3: participants’ ways of coping with workplace stress

The participants’ sources of support and coping mechanisms culminated into four sub-themes. They are individual coping mechanisms, support from colleagues, support from supervisors/social workers, and support from senior management. The sub-themes reflected the social constructionism’s notion that in the pursuit of meaning and understanding of human activities, it is possible to generate differing and sometimes inconsistent explanations of knowledge and reality (Lock & Strong, 2010; Touminen & Savolainen, 1997).

### Sub-theme 3.1: individual coping mechanisms

Some of the participants mentioned that they found their own coping mechanisms being most effective in helping them to deal with work stress. Participants expressed themselves as follows:

When I feel that the work stress is too much, I get home and start spring cleaning … I do a lot of household chores and manual work like washing clothes, bathing the children. Any work that will make me not think about my difficulties with the patients. I keep busy and I then forget about my work problems.

When I experience stress that is difficult to deal with, sometimes I deal with the situation head-on … sometimes I pray and ask God to help me.

Studies among HIV and AIDS lay healthcare workers in the Gauteng Province (South Africa) also found that sometimes care workers became creative with what they could do at an individual level to cope with the work stress. Such coping strategies included art, taking walks, humour and other forms of distraction to relax and rejuvenate (Hatzipapas, 2013; Visser & Mabota, 2015).

### Sub-theme 3.2: support from colleagues

The participants, as HIV and AIDS HBCWs formed part of a team. They were therefore expected to be able to work together, more so that they worked in pairs when doing fieldwork, specifically home-visits. Some of the participants indicated that they valued the support they received from their colleagues. The sentiment was expressed as follows by participants:

When I am working with a patient that has serious difficulties, my colleagues are there to support me, especially those who have been through a similar experience. What they do (work) is what I do so they guide me from experience. It is easier that way.

Sometimes I share my work challenges with my colleagues … especially those that I have a close relationship with … 

Studies in the South African healthcare sector confirmed the value of collegial support. Workers reported dependence on one another for social and interpersonal support that resulted in a boost in morale and feeling of belonging (Hatzipapas, 2013; Rabie, Klopper, & Coetzee, 2017).

### Sub-theme 3.3: support from the supervisors/social workers

Whilst some participants valued individual coping strategies and collegial support, about half of the participants preferred support from either the social worker or supervisor with the social worker being the first preference. Some of the participants put their views as follows:

I explain to the social worker the difficult situation my patients are facing. She would then intervene and work with the family to address the problem. I then wait for her to tell me to do a follow-up visit or something else. I feel better after receiving feedback from the family that they got the help that they needed.

When I am handling a difficult case, once I notify the supervisor, she immediately helps me and sometimes this includes accompanying me to the home visit of the family or patient in question.

Findings in studies among South African lay healthcare workers reiterated the importance of available supervision in the form of emotional and technical support for the workers to enable them to deal with the traumas of HIV work and improve their competence in rendering quality service to their patients (Daniels, Nor, Jackson, Ekstron, & Doherty, 2010; Visser & Mabota, 2015).

### Sub-theme 3.4: support from senior management

A small proportion of participants cited senior management as a source of support. Participants motivated their views by saying:

I feel supported by the Director particularly during monthly meetings that she conducts and asks each one of us individually about our work … how it is progressing and where are we experiencing difficulties. It shows that she cares.

I like the fact that the Director sometimes gives me time to study … sometimes she has meetings with us where she attentively listens when we talk about the problems and challenges we face in our work.

Senior management seemed to form a valuable component of the support structure or team for the lay healthcare workers. Research findings of two South African studies among HIV and AIDS carers emphasised the need among research participants to feel structurally and emotionally supported by senior management and that they found comfort in that kind of support (Daniels et al., 2010; Hatzipapas, 2013).

### Theme 4: participants’ expectations of the stress management programme

Sub-themes were not extracted from this theme as it was important to capture what all participants expressed as their expectations. This stance was informed by social constructionism’s tenet that proposes that in the spirit of critical thinking, all perspectives about social reality have to be entertained as they all matter and are important (Gergen, 2001). The majority of the participants mentioned that they would like to learn about what stress is, how to manage and cope with stress. These expectations were articulated by one participant as follows:

I would like to learn about the types of stress and also other ways of addressing problems so that I get to know what to do. Maybe also learn how to identify signs of stress among children as they do not normally talk about what is bothering them. With the knowledge, I can be able to see immediately and help rather than only find out after months after I had assumed that they are naughty or disobedient.

Other participants shared the following:

I think the programme should help me to deal with stress from the patients especially that is related to HIV and AIDS … I will also be able to help others to manage stress with the information I get.

Sometimes I experience a difficult situation with patients and I need to talk to the social worker immediately. She is not always available. The programme will hopefully help me to learn skills to deal with such situations rather than having to carry that difficulty with me.

I am hoping to understand more information about HIV and AIDS, to be able to share the knowledge with friends, colleagues and my patients. I also expect to learn about how to manage stress so that I do not carry with me and repress the difficulties I am going through.

From the afore-mentioned quotations, it appears that there was a general consensus among the participants and that they expected to learn more about stress and acquire stress management skills. What also needs to be noted is that some did not plan to keep the knowledge only for themselves but also to share with others for whom such information may be beneficial.

### Themes and sub-themes from the post-exposure data

A discussion of the four themes with sub-themes that were extracted from the data post-exposure to the programme follows.

### Theme 1: what participants found fulfilling about their work

Regarding this theme, the same trends as with the pre-intervention data were identified. The sub-themes of sources of job fulfilment that were repeated are opportunities for professional growth and enjoyable work. The majority of the participants still regarded their work as providing opportunities for growth and intellectual stimulation.

The sub-themes are not elaborated on as this was already done with pre-intervention data.

### Theme 2: participants’ perception of challenges in the execution of their work

The sub-themes extracted from this theme were also similar to the pre-intervention sub-themes. The main difference was that after exposure to the programme, the participants elaborated more on the emotional impact of the work and nothing about the lack of skills to deal with challenging patients. Two main sub-themes evolved, namely lack of material resources that affect their ability to meet patients’ needs and experience of the work as emotionally draining. Interestingly, there were a few comments about the inconsistent and inadequate stipend and the uniform.

The two sub-themes are briefly discussed and only supported by participants’ verbatim quotations as they were comprehensively explained in the pre-exposure section.

### Sub-theme 2.1: lack of material resources to meet the patients’ needs

Lack of material resources was again illuminated by the participants as a concern. They also mentioned lack of transport to home-visits as a difficulty (given that the houses are far apart) and limitations with regards to material assistance for their patients, especially food parcels. The majority of the participants raised more concern about the lack of transport which was also the case during pre-exposure interviews. Some participants expressed the group’s sentiments as follows:

I do not like the fact that we have to walk long distances to do home visit. I am also disturbed by the inability to assist clients particularly with material things such as food. There are times when I find my patients’ situation being bad … and they need money to buy food. Remember that we are not allowed to give our patients money but in some situations, it is difficult not to. You then think about the meagre stipend that you receive and you end up not knowing what to do … you need money for yourself and your family … the organisation does not have the food parcels to help.

I find walking long distances in the hot sun from house to house, doing home visits really challenging.

The participants clearly draw attention to service delivery challenges they had to face from time to time and then find ways of coping.

### Sub-theme 2.2: experience of the work as emotionally draining

The participants reflected on what they found emotionally draining after exposure to the stress management programme. Only a few participants mentioned that they still found their work emotionally draining. Their responses are concisely put by some of the participants:

I still feel emotionally affected by my patients’ problems, particularly those that I have been unable to help.

I sometimes find myself feeling physically tired and emotionally drained. The high workload also adds to the challenges that I face.

What the participants seemed to imply was that in spite of having gone through the programme, they still experienced the work as draining particularly with regards to situations they had no control over.

### Theme 3: the participants’ ability to cope with work challenges after exposure to the stress management programme

The participants were asked to give their views with regards to coping after exposure to the stress management programme. Social constructionism proposes that meaning and understanding of social reality is derived from social interaction. It was therefore through the interaction during the intervention process that participants were able to gain insight about and form opinions of what the programme meant to them (Burr, 2003; Freedman & Combs, 1996; Lock & Strong, 2010; Touminen & Savolainen, 1997). All the participants indicated that there was an improvement in their ability to cope. One sub-theme was identified as a result, improvement in coping skills.

### Sub-theme 3.1: improvement in coping skills

The participants mentioned that there was an overall improvement in their ability to cope with work challenges and they attributed this to the programme. A participant expressed her view as follows:

I feel like a load has been taken off my shoulders. My body used to be painful and I did not understand why. After body scan and meditation all the pain was gone. I remembered that I know about meditation but never thought of using it. I was really carrying a lot … meditation was really helpful.

Another said the following:

Sometimes I find myself not coping … but then I start to think about the fact that this situation is beyond my control. I accept the situation as it is, from a realistic point of view. Then I see the problems as manageable.

It appeared that although participants were still faced with work challenges they were coping better than before the programme. Their individual coping strategies seemed to have improved. This finding was supported by two studies (South African & USA), one being in the healthcare sector. The studies found that participants reported marked improvement in their ability to cope with stress after exposure to a brief stress management programme that focused primarily on self-nurturing techniques to manage stress (Crouch, 2008; Pipe et al., 2009).

### Theme 4: participants’ evaluative comments on their experience of the stress management programme

In line with social constructionism encouragement of critical thinking and that there is not one explanation of reality, participants had to critically evaluate the programme and give their views and opinions (Gergen, 2001). From the data gathered, two sub-themes emerged: benefits of the programme and suggestions for improvements to the programme.

### Theme 4.1: benefits of the stress management programme

All the participants mentioned that they benefitted from the stress management programme. Some of their opinions included the following:

I have learnt through the ABCDE (A- activating event; B- belief about the event; C- consequences; D- disputing irrational thoughts; E- new effective rational ways of thinking) model to be patient with my patients even when they are difficult.

… now I can differentiate between different types of stress: good stress and bad stress.

All the modules helped me to cope with stress. I particularly liked the part on resilience a lot.

BREAKS (Background; Rapport; Exploring; Announce; Kindling; Summarising) protocol helped me with ways to approach patients that I meet for the first time it helps breaking the ice. I found all the techniques helpful I also enjoyed the module on the experience of illness. I learnt something new.

I used to make my patient’s problems mine whilst I have my own personal problems. Since the programme, I have managed to create boundaries. I deal with work stress at work. I do not allow my patients’ problems to affect my personal life.

I now know what to do with work stress and I am able to use the techniques to cope.

In support of the participants’ responses, reviews of stress management interventions among healthcare workers found that there was a general improvement in the healthcare workers’ ability to cope with occupational stress and burnout after exposure to individual directed interventions. Furthermore, cognitive–behavioural techniques were found to be most effective followed by relaxation techniques, as compared to other interventions used (Richardson & Rothstein, 2008; Routsalainen, Serra, Marine, & Verbeek, 2008; Siu, Cooper, & Phillips, 2014). An assumption can, therefore, be made that participants found the programme and its interventions helpful.

### Sub-theme 4.2: suggestions for improvements to the stress management programme

The majority of participants explained that they experienced the stress management programme as adequate in empowering them to cope with workplace stress and they would not add or exclude anything from it. They recommended that it be left as is. However, there were a few participants who gave the following suggestions for improvement:

You can maybe have an additional module on self-care. The information that was given during the resilience module was not enough.

I wish you could add information on disabilities. Sometimes in my work, I come across clients that have disabilities and I do not know what to do.

I think it would be a good idea to invite some of the management staff to attend the programme with us, at least the first sessions so that they can experience the programme for themselves and be able to see what it is we are gaining from it. Whoever is available could come: social worker, our supervisor, or even one of the social auxiliary workers.

I suggest that the time allocation for each session be increased particularly for the stress management techniques module … the time was short. It might also be helpful to have two sessions a day rather than one per day.

From the participants’ input on this theme, it appears that there was an overall consensus that the programme met their expectations with a few modifications that were suggested.

After the presentation and discussion of both the pre- and post-exposure data, the two sets of data were compared to identify differences and similarities.

### Comparison of pre- and post-exposure data

In order to further establish whether the stress management programme was effective and efficient in meeting the workplace support needs of the HBCWs, the data pre- and post-exposure to the programme were compared to determine if there were any notable differences. The comparison was done using the themes that were extracted from both sets of data.

### Theme 1: job fulfilment indicators

From the pre-exposure data, the participants indicated that their source of job fulfilment came from opportunities for professional growth that they received, the enjoyment that they derived from the work and lastly the opportunity to help others. The majority of the participants valued the opportunities for professional growth more as compared to the other two.

A similar pattern was observed with the post-exposure data. The only difference was that participants expressed job fulfilment coming mainly from opportunities for professional growth and enjoyment of the work they did. Again, the majority cited professional growth opportunities.

### Theme 2: participants’ perception of challenges in the execution of their work

The participants’ responses, during the pre-exposure phase, highlighted that they were challenged by a lack of material resources and transport to do home visits as well as the insufficient food parcels. The post-exposure data also emphasised the same sentiments that participants shared with regards to lack of resources. Only a few participants indicated that they found their job emotionally draining, mainly due to situations out of their control. An assumption can, therefore, be made that, after exposure to the programme, the participants felt empowered to deal with their work challenges, especially stress-related challenges.

### Theme 3: how participants coped with work stress

Data gathered before the participants were exposed to the programme revealed that the participants used various levels of support to cope with the work stress. They were individual coping mechanisms, support from colleagues, support from supervisors and/or social workers and senior management. As previously indicated, the majority of the participants preferred consulting with the social worker/supervisor the most, followed by individual coping mechanisms and collegial support, and lastly support from senior management.

After exposure to the programme, participants reported that although they still valued the support from the social worker/supervisor, colleagues and senior management, they felt empowered by the intervention in that their individual coping mechanisms have improved and therefore do not need the support as much as they previously did. The comparison of the data collected pre- and post-exposure to the programme, therefore, seem to suggest that there was a noticeable improvement in the participants’ ability to cope with work stress after exposure to the intervention.

### Theme 4: participants’ expectations of the programme versus their experience of the programme

With regards to what the participants expected from the programme, all participants expressed the desire and need to learn something about stress and stress management with an emphasis being on understanding stress and acquiring stress management skills to cope with the work stress they were faced with. Some participants even mentioned that they were planning to share the knowledge and skills with others who were not part of the programme. The post-exposure data confirmed that their expectations of learning about stress and acquiring stress management skills were met as all participants were enthusiastic about what it is they have gained from the programme. A few even gave suggestions on improvements to the programme.

The comparison of data at the pre- and post-exposure levels has demonstrated that the participants’ expectations of the programme were met and they have acquired the necessary stress management skills that are relevant to HIV and AIDS home-based care work.

## Conclusions and recommendations

The participants clearly articulated that the stress management programme was practical and effective in meeting their workplace support needs. The interpretation, comparison and discussion of pre- and post-exposure data, provided valuable feedback on how the participants experienced the programme. Despite the suggestions given on the refinement of the programme, the majority of participants felt that the programme was adequate and does not need any modifications. The main points that were raised by the participants as areas that need improvement were that the time allocation per session be increased particularly with the stress management techniques module; the self-care portion of ‘building resilience’ module should be extended to include additional information on ‘first aid for (the carer’s) mental health’; immediate supervisor and/or management representative should attend the sessions as observers so that they are able to support the HBCWs in maintaining what they have learnt from the intervention programme; and, a request was made to add general information on disabilities as HBCWs sometimes have to work with patients who have disabilities.

The authors are aware that this article only reports on qualitative data which were not corroborated by quantitative results. The data are also not compared to a control group. Another limitation is that the first (lead) author collected data and implemented the programme which could increase the tendency among participants’ to offer favourable responses. Albeit a limitation, numerous strategies were employed to ensure the trustworthiness of the data collection and analysis (see Data collection and analysis). The authors consider the data reported here as valuable to provide input to the advanced development and evaluation of the programme (i.e. Phase 5 of IR) for implementation across the Tshwane Metropolitan area to support a vulnerable occupational group, namely HIV and AIDS HBCWs.

Based on the research findings, recommendations regarding the structure and content of the programme are that the session time allocation is increased from 1 h and 30 min to 3 h because each session took an hour longer on average during the piloting process with a break in between to improve participants’ concentration and participation. The module on ‘building resilience’ (Module 5), be expanded to include additional information on ‘first aid for mental health’ of the HBCWs, with class activities to facilitate practical application. Furthermore, additional class activities for each of the three stress management techniques (CBT, CISD & MBSR, see Table 1) be incorporated for improved integration of theory and practice. A train-the-trainer workshop on the HIV and AIDS HBCWs stress management programme to be made available to the supervisors/managers (of the HBCWs) as a form of capacity building so that they are skilled to facilitate the programme and not rely on the research-practitioner. The final recommendation is that a follow-up evaluation session is held after four months of programme completion, attended by both programme participants and their supervisors/managers to assess the sustainability of the skills acquired during programme implementation to inform future refinement and adjustment of the programme.

Recommendations regarding future research are that a condensed form of the stress management programme with a specific focus on the stress management techniques, be designed and implemented as part of basic home-based care training (e.g. during induction programmes); the programme be adapted for use by other HBCWs who are not working in the HIV and AIDS field; as the programme was implemented with and piloted among HBCWs who worked for NGOs, it is proposed that the programme be adapted and made available to lay healthcare workers employed by government/public health facilities particularly community health clinics. Finally, as the stress management programme was piloted in a predominantly African community, it is suggested that the programme is further piloted with other racial groups within the Tshwane Metropolitan area.

## Appendix. Pre- and post-exposure interview guide

### SECTION A: DEMOGRAPHIC INFORMATION

1. Initials (for identification purposes, only):
2. Age:
3. Gender:
4. Racial group: African /Coloured / White / Asian / Other
5. Marital status: Please mark appropriate box with **X**

**Table:**

Married	Divorced	Widowed	Single	Other(specify)

6. Highest Educational qualifications: Please mark appropriate box with **X**

**Table:**

No schooling	Grade 1 - 7	Grade 8 - 11	Post Grade 12 qualification	Other (specify)

7. Years of experience as a home-based care practitioner:

### SECTION B: INTERVIEW QUESTIONS

**Table:**

Pre-exposure interview questions	Post-exposure interview questions
What do you like about your work? What do you dislike about your work? Do you think your clients/patients value the service that you render? Please explain your answer. I am going to ask you about the emotional impact of your work: 4.1. Are you emotionally affected by the problems of your clients/patients? Please explain your answer. 4.2. If you are affected, how do you cope? Describe what you find challenging about your work. What is your understanding of stress? How do you manage workplace stress? **Complete the following sentences:** 8. I feel supported by my manager/supervisor when ……… 9. I feel supported by my colleague/s when ……… 10. I feel supported by my organisation/employer when ……… 11. My expectations of a stress management programme are ……… 12. What are your professional goals for the next two years (where do you see yourself as a home-based care practitioner in the next two years)?	What do you like about your work? What do you dislike about your work? Do you think your clients/patients value the service that you render? Please explain your answer. I am going to ask you about the emotional impact of your work: 4.1. Are you emotionally affected by the problems of your clients/patients since you have been through the stress management programme? Please explain your answer. 4.2. If you are affected, how do you cope? Describe what you find challenging about your work. 5.1. How do you cope with the challenges? **Complete the following sentences:** 6. I have experienced the stress management programme as helpful because ……… 7. I have experienced the stress management programme as unhelpful because ……… 8. I suggest that the following should be excluded from the stress management programme in future 9. I suggest that the following should be included in the stress management programme in future

## References

[CIT0001] Akintola, O. (2011). What motivates people to volunteer? The case of volunteer AIDS caregivers in faith-based organizations in KwaZulu-Natal, South Africa. *Health Policy & Planning*, *26*, 53–62. doi: 10.1093/heapol/czq019 20511348

[CIT0002] Andrews, T. (2012). What is social constructionism? *Grounded Theory Review*, *1*(1), 39–46. Retrieved from http://www.groundedtheoryreview.com

[CIT0003] Babbie, E., & Mouton, J. (2011). *The practice of social research*. Cape Town: Oxford University Press.

[CIT0004] Botha, R. (2002). Using an adventure medium to construct unique outcomes: A social constructionist approach (Unpublished master’s dissertation). University of Pretoria, Pretoria.

[CIT0005] Braun, V., & Clarke, V. (2013). *Successful qualitative research: A practical guide for beginners*. Thousand Oaks, CA: Sage.

[CIT0006] Burr, V. (2003). *Social constructionism* (2nd ed.). London: Routledge.

[CIT0007] Campbell, C., & Foulis, C. (2004). Creating context for effective home-based care of people living with HIV/AIDS. *Curationis*, *27*(3), 5–14. Retrieved from http://eprints.lse.ac.uk doi: 10.4102/curationis.v27i3.993 15777025

[CIT0008] Campbell, C., Nair, Y., Maimane, S., & Sibiya, Z. (2008). Supporting people with AIDS and their carers in rural South Africa: Possibilities and challenges. *Health and Place*, *14*, 507–518. doi: 10.1016/j.healthplace.2007.10.002 18023238

[CIT0009] Campbell, S. (2014). What is qualitative research? *American Society for Clinical Laboratory Science*, *27*(1), 3. Retrieved from http://www.search.proquest.com doi: 10.29074/ascls.27.1.3 24669439

[CIT0010] Choudhury, K. (2013). *Managing workplace stress: The cognitive behavioural way*. Kolkata: Springer.

[CIT0011] Cogan, L., Klein, M., Magongo, R. G., & Kganakga, M. C. (2005). *The psychosocial aspects of HIV/AIDS: Adults.* Retrieved from http//www.bayloraids.org/curriculum/files

[CIT0012] Colligan, T. W., & Higgins, E. M. (2006). Workplace stress. *Journal of Workplace Behavioural Health*, *21*, 89–97. doi: 10.1300/J490v21n02_07

[CIT0013] Crouch, R. B. (2008). A community-based stress management programme for an impoverished population in South Africa. *Occupational Therapy International*, *15*, 71–86. doi: 10.1002/oti.246 18278826

[CIT0014] Cunliffe, A. L. (2008). Orientations to social constructionism: Relationally responsive social constructionism. *Management Learning*, *39*, 123–139. doi: 10.1177/1350507607087578

[CIT0015] Dageid, W., Sedumedi, S. D., & Durkert, F. (2007, September). Exploring perceptions and emotional burdens of HIV/AIDS health care workers. Paper presented at the Human Sciences Research Council Conference, Benoni, South Africa.

[CIT0016] Daniels, K., Nor, B., Jackson, D., Ekstron, E., & Doherty, T. (2010). Supervision of community peer counsellors for infant feeding in South Africa: An exploratory qualitative study. *Human Resources for Health*, *8*, 6–15. doi: 10.1186/1478-4491-8-6 20353561 PMC2867794

[CIT0017] Department of Social Development. (2007). *National norms and minimum standards for home and community-based care and support programme*. Pretoria: Government Printers.

[CIT0018] Department of Social Development, Department of Education & Department of Health. (2004). *Expanded public works programme: social sector plan 2004/5–2008/9.* Retrieved from http://www.labour.org.za

[CIT0019] De Wet, K., & Du Plooy, S. (2012). “We are left in the cold:” Nurses perceptions and responses to antiretroviral treatment roll-out in the Free State, South Africa. *SAHARA-J: Journal of Social Aspects of HIV/AIDS*, *9*, 30–40. doi: 10.1080/17290376.2012.665256 23237019

[CIT0020] Du Preez, C., & Niehof, A. (2008). Caring for people with AIDS: A labour of love. *Medische Antropologie*, *20*(1), 87–104. Retrieved from http://www.medanthrotheory.org

[CIT0021] Etikan, I., Musa, S. A., & Alkassim, R. S. (2016). Comparison of convenience sampling and purposive sampling. *American Journal of Theoretical and Applied Statistics*, *5*(1), 1–4. doi: 10.11648/j.ajtas.20160501.11

[CIT0022] Fouché, C. B., & Schurink, W. (2011). Qualitative research design. In A. S. de Vos, H. Strydom, C. B. Fouché, & C. S. L. Delport (Eds.), *Research at grassroots: For social sciences and human service professions* (4th ed., pp. 307–327). Pretoria: Van Schaik.

[CIT0023] Freedman, J., & Combs, G. (1996). *Narrative therapy: The social constructionism of preferred realities*. New York, NY: W. W. Norton & Company.

[CIT0024] Gergen, K. J. (2001). *An invitation to social construction*. London: Sage.

[CIT0025] Geteri, L. M., & Angogo, E. M. (2013). Self-care among caregivers of people living with HIV and AIDS in Kakola location, Nyando District, Kisumu County, Kenya. *SAHARA-J: Journal of Social Aspects of HIV/AIDS*, *10*, 65–71. doi: 10.1080/17290376.2013.807065 PMC3914458 24090080

[CIT0026] Greeff, M. (2011). Information collection: Interviewing. In A. S. de Vos, H. Strydom, C. B. Fouché, & C. S. L. Delport (Eds.), *Research at grassroots: For social sciences and human service professions* (4th ed., pp. 341–375). Pretoria: Van Schaik.

[CIT0027] Greenspan, J. A., McMahon, S. A., Chebet, J. J., Mpunga, M., Urassa, D. P., & Winch, P. J. (2013). Sources of community health worker motivation: A qualitative study in Morogoro Region, Tanzania. *Human Resources for Health*, *11*, 52. 10.1186/1478-4491-11-52 24112292 PMC3852396

[CIT0028] Gregory, R., & Thorley, L. (2013). *Using group-based learning in higher education*. London: Kogan Page.

[CIT0029] Haski-Levennthal, D. (2009). Altruism and volunteerism: The perceptions of altruism in four disciplines and their impact on the study of volunteerism. *Journal for the Theory of Social Behaviour*, *39*, 271–299. doi:10.2989/16085906.2015.1040812 doi: 10.2989/16085906.2015.1040812

[CIT0030] Hatzipapas, I. (2013). Exploring experiences of care-workers participating in laughter therapy (Unpublished master’s dissertation). University of Pretoria, Pretoria.

[CIT0031] Heunis, J. C., Wouters, E., & Kigozi, N. G. (2012). HIV, AIDS and tuberculosis in South Africa: Trends, challenges and responses. In H. C. J. van Rensburg (Ed.), *Health and health care in South Africa* (2nd ed., pp. 284–360). Pretoria: Van Schaik.

[CIT0032] Hlophe, H. (2006). Home-based care as an indispensable extension of professional care in ART – a plea for recognition and support. *Acta Academia Supplementum*, *2006*(1), 191–215. Retrieved from http://journals.ufs.ac.za/index.php/aa/article/download/1109/1098

[CIT0033] Joint United Nations Programme for HIV/AIDS. HIV/AIDS fact sheet July, 2018. (2018). Retrieved from http://www.unaids.org

[CIT0034] Joint United Nations Programme for HIV/AIDS - South Africa, 2018. (2018). Retrieved from http://www.unaids.org

[CIT0035] Jordan, J. R. (2011). Groupwork with suicide survivors. In J. R. Jordan & J. L. McIntosh (Eds.), *Grief after suicide: Understanding the consequences and caring for the survivors* (pp. 283–296). New York, NY: Routledge.

[CIT0036] Kabamba, T. L. (2009). Psychosocial challenges facing HIV/AIDS lay counsellors at a community-based voluntary counselling and testing site in Tshwane (Unpublished master’s mini-dissertation). University of South Africa, Pretoria.

[CIT0037] Kupa, P. M. (2019). *A stress management programme for HIV and AIDS home-based carepractitioners in Tshwane* (Unpublished doctoral thesis). University of Pretoria, Pretoria.

[CIT0038] Lehmann, U., & Sanders, D. (2007). *Community health workers: What do we know about them?* Cape Town: University of Western Cape.

[CIT0039] Lekganyane, M. R., & Alpaslan, A. H. (2019). Suggestions by home-based caregivers caring for people living with HIV and AIDS on how social workers could support them in managing work-related challenges. *Social Work/Maatskaplike Werk*, *55*(2), 141–157. doi:10.15270/52-2-712 doi: 10.15270/52-2-712

[CIT0040] Lietz, C. A., & Zayas, L. E. (2010). Evaluating qualitative research for social work practitioners. *Advances in Social Work*, *11*(2), 188–202. Retrieved from http://journals.iupui.edu/index.php/advancesinsocialwork/article/view/589 doi: 10.18060/589

[CIT0041] Linganiso, W. S., & Gwegweni, J. M. T. (2016). What perpetuates the spread of HIV/AIDS in rural South African communities? A closer look at social factors. *Austin Journal of Public Health and Epidemiology*, *3*(1), 1031. Retrieved from http://www.austinpublishing.com

[CIT0042] Lock, A., & Strong, T. (2010). *Social constructionism: Source and stirrings in theory and practice*. Cambridge: Cambridge University Press.

[CIT0043] Lund, F. (2010). Hierarchies of care work in South Africa: Nurses, social workers and home-based care workers. *International Labour Review*, *149*(4), 495–509. Retrieved from http://unrisd.org doi: 10.1111/j.1564-913X.2010.00100.x

[CIT0044] Lund, F., & Budlender, D. (2009). *Paid care providers in South Africa: Nurses, domestic workers and home-based care workers. Research Report 4*. Geneva: UNRISD. Retrieved from http://www.unrisd.org

[CIT0045] Majaja, M., Setswe, G., Davids, A., Clayton, J., Naidoo, Y., Lewa, M., & Simbayi, L. (2009, September). Psychosocial support (PSS) needs of community home-based carers (CHBC) working with orphans and people living with HIV/AIDS (PLWA) in South Africa: Case of Red Cross Society. Paper presented at AIDS Impact Conference, Gaborone, Botswana.

[CIT0046] Marais, H. (2005). *Buckling: The impact of AIDS in South Africa*. Pretoria: University of Pretoria.

[CIT0047] Narayanan, V., Bista, B., & Koshy, C. (2010). ‘BREAKS’ protocol for breaking bad news. *Indian Journal of Palliative Care*, *16*(1), 61–65. doi: 10.4103/0973-1075.68401 21811349 PMC3144432

[CIT0048] National Planning Commission. (2011). *National development plan 2030.* Retrieved from http://npconline.co.za

[CIT0049] Ntobeng, S. N. (2016). The psychosocial circumstances of community care givers: A case of Tshwane Region (Unpublished master’s mini-dissertation). University of the Witwatersrand, Johannesburg.

[CIT0050] Okeke, B. O. (2016). Social support seeking and self-efficacy-building strategies in enhancing the emotional well-being of informal HIV/AIDS caregivers in Ibadan, Oyo state, Nigeria. *SAHARA-J: Journal of Social Aspects of HIV/AIDS*, *13*, 35–40. doi: 10.1080/17290376.2015.1126794 PMC5642436 26831832

[CIT0051] Orner, P. (2006). Psychosocial impacts on caregivers of people living with AIDS. *AIDS Care*, *18*, 236–240. doi: 10.1080/09540120500456565 16546784

[CIT0052] Padgett, D. K. (2017). *Qualitative methods in social work research* (3rd ed.). New York, NY: Sage.

[CIT0053] Pienaar, M., Van Rooyen, F. C., & Walsh, C. M. (2017). Household food security and HIV status in rural and urban communities in the Free State province, South Africa. *SAHARA-J: Journal of Social Aspects of HIV/AIDS*, *14*(1), 118–131. doi: 10.1080/17290376.2017.1379428 PMC5639613 29020850

[CIT0054] Pipe, T. B., Bortz, J. J., Dueck, A., Pendergast, D., Buchda, V., & Summers, J. (2009). Nurse leader mindfulness meditation program for stress management: A randomized trial. *JONA: The Journal of Nursing Administration*, *39*, 130–137. doi: 10.1097/NNA.0b013e31819894a0 19590469

[CIT0055] Rabie, T., Klopper, H. C., & Coetzee, S. K. (2017). Creating positive practice environments in a primary healthcare setting. *International Journal of Nursing Practice*, *2017*, 1–8. doi: 10.1111/ijn.12555 28556407

[CIT0056] Richardson, K. M., & Rothstein, H. R. (2008). Effects of occupational stress management intervention programs: A meta-analysis. *Journal of Occupational Health Psychology*, *13*, 69–93. doi: 10.1037/1076-8998.13.1.69 18211170

[CIT0057] Rohleder, P., & Swartz, L. (2005). What I’ve noticed is that what they need is the stats’: Lay HIV counsellors’ reports of working in a task-orientated healthcare system. *AIDS Care*, *17*, 397–406. doi: 10.1080/09540120512331314376 15832888

[CIT0058] Routsalainen, J., Serra, C., Marine, A., & Verbeek, J. (2008). Systematic review of interventions for reducing occupational stress in health care workers. *Scandinavian Journal of Work, Environment & Health*, *34*(3), 167–178. Retrieved from http://www.jstor.org/stable/40967705 10.5271/sjweh.1240 18728906

[CIT0059] Schneider, H., Hlophe, H., & Van Rensburg, D. (2008). Community health workers and the response to HIV/AIDS in South Africa: Tensions and prospects. *Health Policy and Planning*, *23*, 179–187. doi: 10.1093/heapol/czn006 18388133

[CIT0060] Sharma, G. (2017). Pros and cons of different sampling techniques. *International Journal of Applied Research*, *3*(7), 749–752. Retrieved from http://www.allresearchjournal.com

[CIT0061] Shenton, A. K. (2004). Strategies for ensuring trustworthiness in qualitative research projects. *Education for Information*, *22*, 63–75. doi: 10.3233/EFI-2004-22201

[CIT0062] Siu, O. L., Cooper, C. L., & Phillips, D. R. (2014). Intervention studies on enhancing work well-being, reducing burnout and improving recovery experiences among Hong Kong health care workers and teachers. *International Journal of Stress Management*, *21*, 69–84. doi: 10.1037/a0033291

[CIT0063] Tanyi, P. L., Pelser, A., & Okeibunor, J. (2018). HIV/AIDS and older adults in Cameroon: Emerging issues and implications for caregiving and policy-making. *SAHARA-J: Journal of Social Aspects of HIV/AIDS*, *15*, 7–19. doi: 10.1080/17290376.2018.1433059 PMC5804678 29409392

[CIT0064] Thomas, E. J., & Rothman, J. (1994). An integrative perspective on intervention research. In J. Rothman & E. J. Thomas (Eds.), *Intervention research: Design and development for human service* (pp. 1–20). New York, NY: Haworth Press.

[CIT0065] Touminen, K., & Savolainen, R. (1997). *A social constructionist approach to the study of information use as a discursive action*. London: Taylor Graham Publishing.

[CIT0066] Visser, M., & Mabota, P. (2015). The emotional wellbeing of lay HIV counselling and testing counsellors. *African Journal of AIDS Research*, *14*, 169–177. doi: 10.2989/16085906.2015.1040812 26223334

[CIT0067] Welcome to EPWP. (2013). Retrieved from http://www.epwp.gov.za

[CIT0068] Wheelan, S. A. (2009). Group size, group development and group productivity. *Small Group Research*, *40*, 247–262. doi:1077/1046496408328703 doi: 10.1177/1046496408328703

[CIT0069] Wringe, A., Cataldo, F., Stevenson, N., & Fakoya, A. (2010). Delivering comprehensive home-based care programmes for HIV: A review of lessons learned and challenges ahead in the era of antiretroviral therapy. *Health Policy and Planning*, *25*(1), 352–362. doi: 10.1093/heapol/czq005 20144935

